# Susceptibility to ***Aspergillus*** Infections in Rats with Chronic Obstructive Pulmonary Disease via Deficiency Function of Alveolar Macrophages and Impaired Activation of TLR2

**DOI:** 10.1007/s10753-016-0363-x

**Published:** 2016-06-16

**Authors:** Yuting Wu, Hong Xu, Li Li, Weifeng Yuan, Deming Zhang, Wenjie Huang

**Affiliations:** Graduate School, Southern Medical University, No1023, Shatai South Street, Guangzhou, Guangdong 510515 China; Respiratory Center of PLA, General Hospital of Guangzhou Command of PLA, Gangzhou, Guangdong 510010 China; Department of elderly Respiratory, General Hospital of Guangzhou Command of PLA, Guangzhou, Guangdong 510010 China

**Keywords:** chronic obstructive pulmonary disease, *Aspergillus*, alveolar macrophages, TLR2

## Abstract

Clinical evidence indicates that patients with severe chronic obstructive pulmonary disease (COPD) are more susceptible to *Aspergillus*. However, the exact mechanisms underlying this effect are not known. In this study, we used cigarette smoke exposure to generate COPD rat model. colony-forming units (CFU) count assessment and phagocytosis were applied to evaluate the defense function of COPD rats against *Aspergillus* challenge. ELISA, western blotting, and GST-Rac1 pull-down assays were conducted to determine the expressions of cytokines and TLR2-associated signaling pathway. Our data showed that *Aspergillus* burdens increased, phagocytosis of *Aspergillus* as well as the expressions of inflammatory cytokines from alveolar macrophages (AMs) were impaired in COPD rats compared with normal rats. Though TLR2 signaling-related proteins were induced in response to the stimulation of *Aspergillus* or Pam3csk4 (TLR2 agonist), the activation of TLR2-associated signaling pathway was apparently interfered in rats with COPD, compared to that in normal rats. Taken together, our study demonstrated that COPD caused the deficiency of AMs function and impaired the activation of TLR2/PI3K/Rac 1 signaling pathway, leading to invasion of *Aspergillus* infection, which also provides a future basis for the infection control in COPD patients.

## INTRODUCTION

Invasive pulmonary aspergillosis (IPA) is caused by *Aspergillus* and characterized as a life-threatening pneumonia due to lung parenchyma invasion with vasculature erosion and necrosis. Growing evidence suggests that the incidence of IPA is correlated with the increase of chronic obstructive pulmonary disease (COPD) [1–3].

Under normal circumstances, *Aspergillus* species are widely distributed in nature and have no invasion to immunocompetent individuals owing to host immune defense system. Of note, respiratory mucosal epithelial cells serve as an anatomic barrier to parenchymal invasion, promote mucociliary clearance, and ingest inhaled conidia [4]. Alveolar macrophages (AMs) consist of multiple kinds of immune cells within the alveolar space and act as a first line of innate host defense against inhaled conidia [5], which employ an array of receptors to recognize pathogen-associated molecular patterns (PAMPs) and to facilitate phagocytic uptake [6].

Cigarette smoking poses as the major risk factor for COPD and accounts for approximately 85 % of all cases. About 15 % of smokers will develop COPD, whereas the incidence in nonsmokers is 1.6 % [7]. Exposure to cigarette smoking markedly impairs lung immunity, resulting in mucociliary dysfunction, mucus hypersecretion, disturbance of the mucosal integrity [8], and easy colonization of *t* potentially pathological microorganisms in lung. Assay of bronchoalveolar lavage fluid (BALF) showed that 90–95 % of cells were from AMs with properties of highly phagocytic, production of multiple inflammatory mediators. Moreover, their role in removal of potentially pathogenic microorganisms via phagocytosis is essential in the maintenance of the normally sterile environment within the lung. One reason for the increased incidence of bacterial infections in the respiratory tract of COPD patients might be failure of macrophages to clear pathogens [9–11]. However, mechanism concerning the effect of cigarette smoking at the interaction between *Aspergillus* and AMs remains to be further understood.

In the present study, we investigated the features of the *Aspergillus* clearance in rat models of smoke-induced chronic obstructive pulmonary disease. Furthermore, we analyzed the impact of cigarette smoke on the phagocytosis of AMs to *Aspergillus* conidia, and cytokine expression as well as *Aspergillus* defense-related receptor on AMs was also studied.

## MATERIALS AND METHODS

### Animals

Female Wistar rats weighing 230–250 g were purchased from Guangdong Laboratory Animal Center (Guangzhou, China). The rats were housed under specific pathogen-free conditions and maintained on a 12-h light–dark cycle, with food and water *ad libitum*. All experiments were approved by Southern Medical University Animal Investigational Committee and were performed in accordance with the Guide for the Care and Use of Laboratory Animals published by the Ministry of Health of China.

### Cigarette Smoke Exposure

Cigarette smoke exposure may be used to create COPD model [12]. Rats were placed in a 110 cm × 86 cm × 72 cm chamber and exposed to cigarette smoke generated by cigarette smoking machine (Kangda Enterprises, Guangzhou, China). Ten cigarettes (Cocopalm, Guangzhou cigarette factory, tar concentration 12 mg) were burned in 8 min, and the rats stayed in the chamber for 45 min each time. Animals were exposed twice/day, 6 day/week, for 12 weeks. Control animals were housed in an identical chamber, exposed to room air without smoke.

### Preparation and Administration of *A. fumigatus* Conidia

A strain of *A. fumigatus* isolated from a patient with invasive aspergillosis was used in this study. To prepare the inoculum, *A. fumigatus* grew on Sabouraud dextrose agar plates for 2 weeks at 37 °C. Conidia were collected by washing the plates with sterile phosphate-buffered saline containing 0.2 % (vol/vol) Tween 80. The conidia were concentrated by centrifugation and determined using a hemacytometer. Rats were intratracheally inoculated with a single administration of 1 × 10^7^ conidia of *A. fumigatus* in 0.2 ml of sterile saline [13].

### Colony-Forming Units (CFU) Count Assessment

Fungal burdens in the lungs were determined by CFU counting. Rats were sacrificed at selected time points (1, 3, 5, 7, and 14 days) after exposure. One gram of lung tissue was aseptically removed and homogenized with an overhead RW16 Basic S1 Overhead Stirrer (IKA Works Inc., Wilmington NC) in 9 ml of sterile saline with gentamicin (0.025 g/l; Sigma) and chloramphenicol (0.4 g/l; Sigma). Primary homogenate dilutions were quantitatively cultured by serial dilution, plated on PDA plates, and incubated at 37 °C for 24 to 36 h, after *A. fumigatus* fungal burdens (numbers of CFU per gr of lung tissue) were determined [14].

### Bronchoalveolar Lavage (BAL) and AMs Culture

Lungs were lavaged through an intratracheal cannula with calcium- and magnesium-free PBS supplemented with 0.6 mM EDTA. A total of 20 ml was used in each rat in 0.5-ml increments with a dwell-time of 30 s. The cells from the lavage fluids were collected by centrifugation 300×*g* for 10 min at room temperature. Isolated cells were washed with PBS, counted using a hemacytometer, and cultivated on glass cover slips in RPMI1640 medium supplemented with 10 % heat-inactivated fetal calf serum, 2-mM glutamine, and penicillin-streptomycin. The cells were transferred into six well plates followed by the 2-h incubation with 5 % CO_2_ at 37 °C. Histological staining and immunofluorescence analysis using CD68 revealed that the vast majority of the adherent cells derived from the alveolar lavages were macrophages.

### Phagocytosis

The biotin-calcofluor staining was performed as previously described [15]. Briefly, swollen conidia were biotinylated using 10 mg/ml sulfo-NHS-LC-biotin (Sigma) in 50 mM NaHCO_3_, pH 8.5 for 2 h at 4 °C. Remaining reactive biotin molecules were inactivated by incubation in 100-mM Tris–HCl, pH 8.0 for 40 min at 4 °C. For phagocytosis experiments, macrophages were seeded on glass cover slips in six well plates at a density of 1 × 10^6^ cells per well. Four hours later, the supernatant was collected and stored at −70 °C for further use. Then, 1-ml warm RPMI 1640 was supplemented, and 5 × 10^6^ biotinylated conidia were seeded at each well. After 1 h at 37 °C and 5 % CO_2_, the supernatant was removed and stored at −70 °C after centrifugation. The glass cover slips were fixed in 3.7 % formaldehyde in PBS for 10 min. Extracellular conidia were detected using Cy3-labeled streptavidin (Sigma) (diluted 1: 100 in PBS, 30 min at 37 °C). Extra and intracellular conidia were stained using 0.4 mg/ml calcofluor white (Sigma) in PBS for 30 min. Macrophages were visualized using 0.1 mg/ml FITC-labeled concanavalin A (Sigma) in PBS for 30 min at room temperature (RT). Micrographs taken with Olympus DP72 RBE microscope (Leica Microsystems, Wetzlar, Germany) and digitally recorded using the MetaMorph software (Visitron Systems, Puchheim, Germany) were used for quantitative analysis. Efficiency of phagocytosis was calculated as phagocytic index and phagocytic rate.

### ELISA

Cytokine levels (TNFα, MIP-2, IL-1β, IL-10) in the supernatants were determined with the commercially available ELISA kit (R&D Systems Inc. Chicago, IL, USA) according to the manufacturer’s instructions.

### Western Blot

The alveolar lavage was performed in randomly selected COPD group and normal control group (each group consisted of three rats), respectively. After treating cells as described above (centrifugation and resuspension), the cells were transferred into two 25-cm^2^ culture flasks followed by the 2-h incubation with 5 % CO_2_ at 37 °C. After washing with PBS for three times, we stimulated the AMs in the COPD group and normal control group for 2 h by using *A. fumigatus* conidia or TLR2 agonist Pam3csk4 (Tocris Bioscience). Total proteins samples were extracted from AMs using lyses buffer containing phenylmethyl sulfonyfluoride (PMSF) supplemented with protease inhibitor cocktail (Roche) and phosphatase inhibitor PhosSTOP (Roche). The samples were mixed with loading buffer and denatured, separated by electrophoresis in a 10 % SDS-PAGE gel, and then transferred to PVDF membranes. The membranes were blocked with 5 % skim milk for 1 h, incubated with anti-TLR2, anti-Rac1 antibody (all from Abcam, Cambridge, UK), anti-AKT, anti-p-AKT, anti-GAPDH antibody (all from CST, USA) at 4 °C overnight. Signals were revealed after incubation with anti-rabbit IgG secondary antibody (CST, USA) coupled to peroxidase by using ECL.

### GST- Rac1 Pull-down Assays

Cells were washed in ice-cold PBS rapidly and lysed on ice in 50-mM Tris–HCl (pH 7.4), 1 % Triton X-100, 10 % glycerol, 2 mMMgCl_2_, 100 mM NaCl, and protein inhibitor mixture. Lysates were centrifuged at 17,000×*g* at 4 °C for 5 min, and samples were taken from the supernatant to estimate total protein concentration. Twenty microgram of GST-Rac1 fusion bound to sepharose beads were added to cell lysate and incubated at 4 °C for 30 min. Beads were washed in lysis buffer four times, and bound proteins were eluted in Laemmli sample buffer. Total proteins and Rac1 affinity-purified proteins were analyzed by Western blotting using anti-Rac1 antibody (Abcam, Cambridge, UK).

### Statistical Analysis

Data are presented as mean ± SD. Statistical analyses were performed using the SPSS version 19.0 software. For parametric data, mean values were compared using a Student’s *t* test. Analysis of variance (ANOVA) was used to compare mean values for the different groups, followed by Student–Newman–Keuls test for multiple comparisons. A *P* value <0.05 was accepted as statistically significant.

## RESULTS

### Comparison of Pulmonary Fungal Burdens

**Fig. 1:**
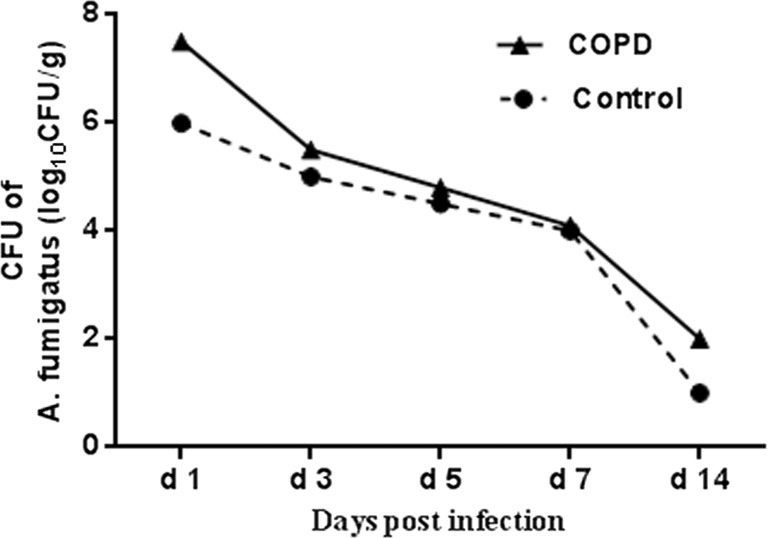
CFU count assessment in each group. The analysis of variance with factorial design showed that difference between normal and COPD groups was significant (*P* < 0.001). The *Aspergillus* burdens in COPD group were significantly higher than those in control at each time point except day 7 (*P* < 0.05).

Quantitative culture analysis showed that the burdens of *Aspergillus* in the lungs were gradually reduced in all the animals challenged with *Aspergillus* spores. Of note, the *Aspergillus* burdens in COPD group were significantly higher than those in the control group at each time point except day 7 (Fig. 1), and no invasive infections by *Aspergillus* were observed in the liver, spleen, kidney, and pancreas tissue in each group.

### Phagocytosis of *A. fumigatus* in AMs

**Fig. 2:**
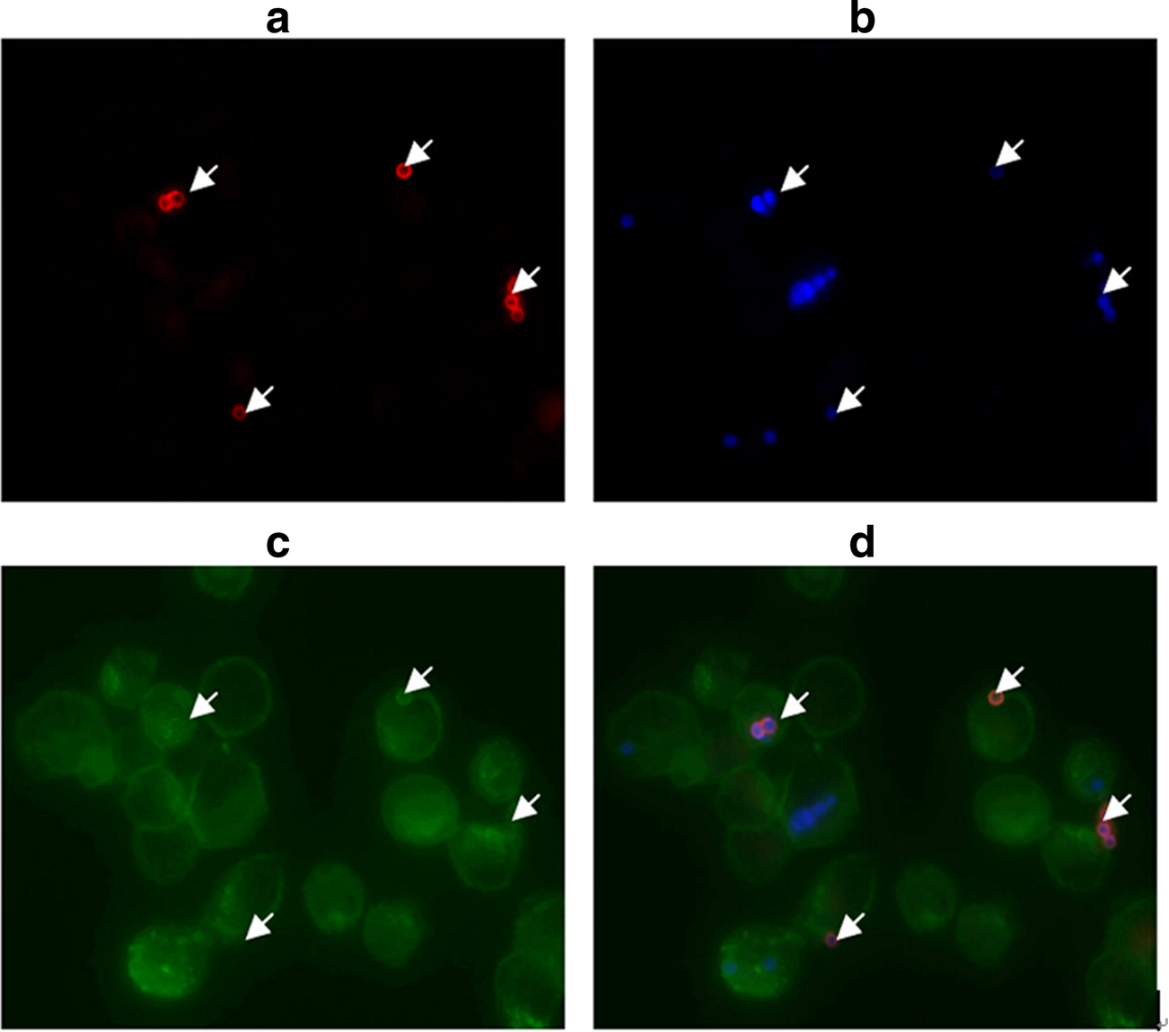
BCS staining of AMs infected with biotinylated. ***A*** *fumigatus* conidia. Differential staining of extra and intracellular conidia. **a** Extracellular conidia stained in *red* using Cy3-labeled streptavidin; **b** calcofluor white staining (*blue*) of extra and intracellular conidia; **c** macrophages visualized by Concanavalin A-FITC are shown in *green* (Concanavalin A-FITC also recognizes the surface of extracellular conidia.) **d** an overlay of all micrographs. Positions of extracellular conidia are indicated by *arrows*.

The result of immunofluorescence stain showed a clear distinction between extra and intracellular conidia (Fig. 2). In addition, we found that cigarette smoke exposure impaired the phagocytosis of *A. fumigatus* in AMs. The phagocytic rate in COPD group was much lower than that in normal group (25.8 ± 4.2 % *vs*. 50.6 ± 14.7 %, *P* = 0.048), but no significant change was observed in phagocytic index (2.5 ± 0.8 *vs*. 3.1 ± 0.8, *P* = 0.38).

### Cytokine Expression in COPD Rats

**Fig. 3:**
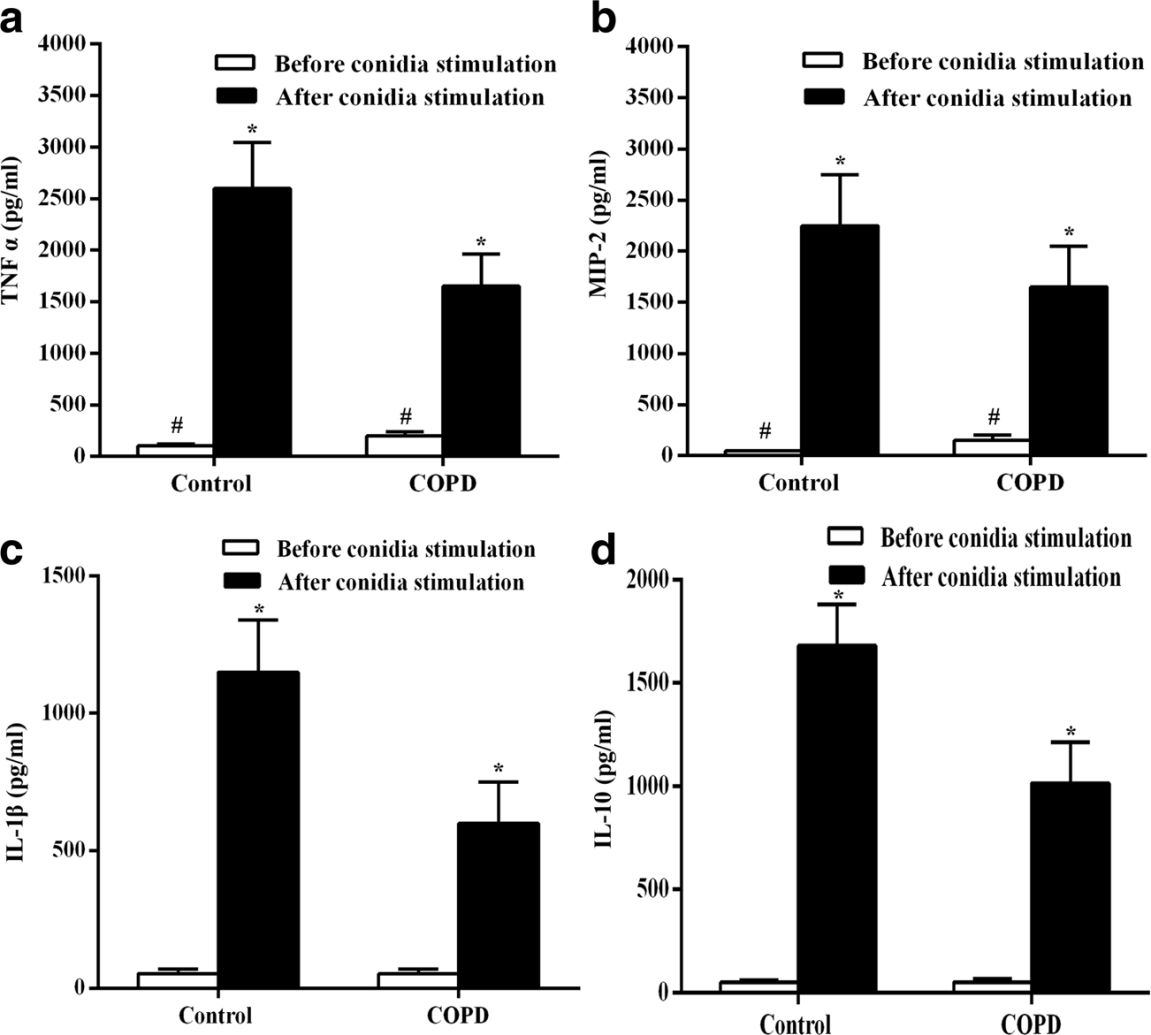
Cytokine levels in supernatant before and after conidia stimulation. The analysis of variance with factorial design showed differences between control and COPD groups. There was interaction between grouping and conidia stimulation, suggesting that the alteration trend of TNF-α, MIP-2, IL-1β, and IL-10 in the culture supernatant after stimulation was different between the groups. In control group, cytokine levels increased more significantly. *Number sign* signifies the analysis of single factor effect which showed that before conidia stimulation, levels of TNF-α and MIP-2 in COPD group were significantly higher than those in control group (*P* < 0.001). *Asterisk* signifies after spore stimulation, although cytokine concentrations in both groups increased significantly, the final cytokine levels were significantly higher in control group than in COPD group (*P* < 0.001).

Slight increasing expressions of TNF-α, MIP-2 were found in COPD rats compared to normal ones. Notably, after conidia challenge, levels of TNF-α, MIP-2, IL-1β, and IL-10 were significantly upregulated, indicating an innate defense of host against *Aspergillus*. However, the growing levels of cytokines in COPD rats were lower than those in normal rats, manifesting impaired defense function in rats with COPD (Fig. 3).

### Expression of TLR2 on AMs of COPD Rats

**Fig. 4:**
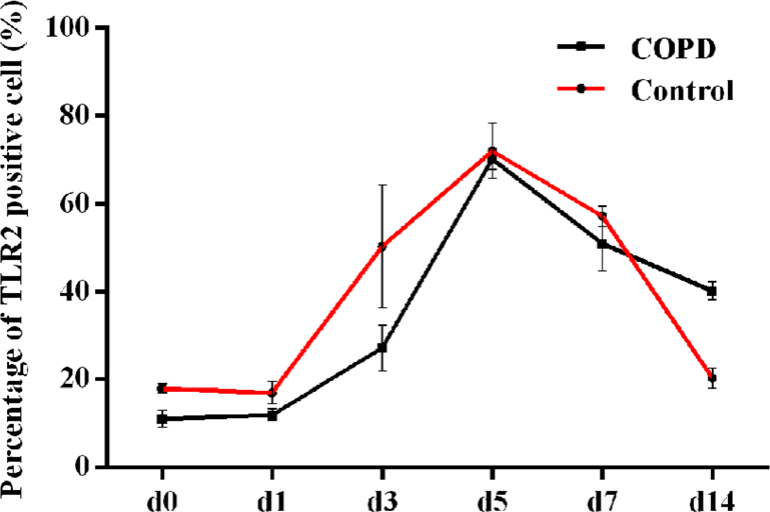
Expressions of TLR2 on AMs after intratracheal instillation of *Aspergillus* spores in each group. The analysis of variance with factorial design showed significant difference between normal and COPD groups (*P* = 0.049), and there were significant differences between different day points (*P* < 0.001). There was interaction between grouping and day points, suggesting that the alteration trends of TLR2 receptor cells in two groups were different.

After the challenge of *Aspergillus* spores, the expression of TLR2 receptors on AMs was increased in two groups and reached a peak on day 5. Moreover, we detected unequal trend of the change in the percentage of TLR2-positive cells in two groups. Particularly on day 3 after infection, TLR2 expression in COPD rats did not show as high level as that in normal rats (Fig. 4). This suggested that the upregulation of TLR2 expression on AM might be delayed in COPD rats.

### *Aspergillus* fumigates Impairs the Activation of TLR2/Akt/Rac1 Pathway

**Fig. 5:**
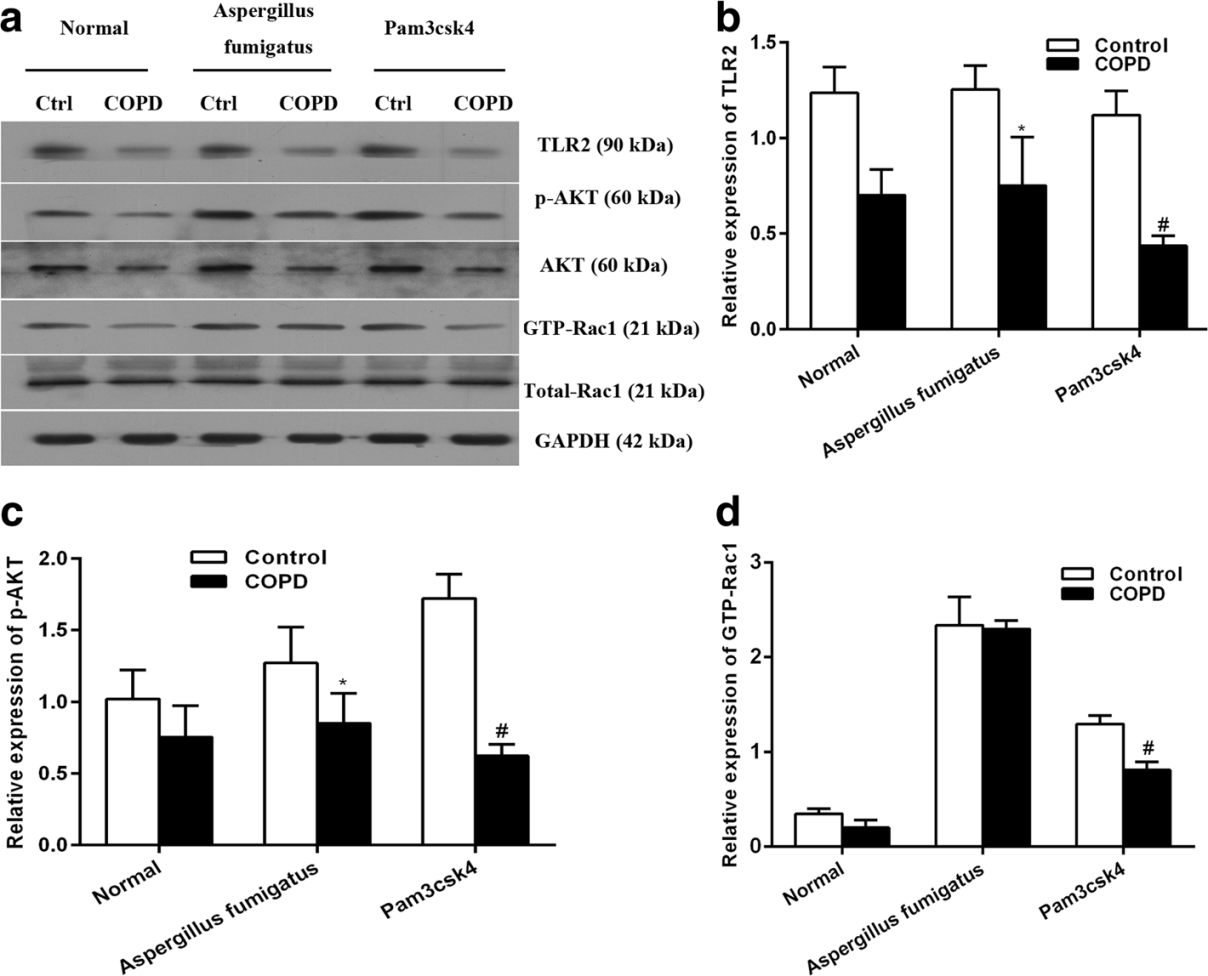
*A. fumigatus* stimulates TLR2/Akt activity through a Rac1-dependent mechanism. **a** Representative image of the western blot results, which showed that the TLR2 protein expression level was downregulated in the COPD rats comparing to the control after *A. fumigatus* infected. These results have also been gotten from the protein expression levels of p-AKT and GTP-Rac1. There were no significant differences found in the AKT and total-Rac1 protein expression levels between the COPD and control groups. **b**–**d** Statistical analysis of the western blot results (*P < 0.05, #*P* < 0.01).

To test the TLR2 signaling pathway in rat alveolar macrophages, we treated rat alveolar macrophages of COPD model and control rats with the treatment of *Aspergillus* or Pam3csk4 for 2 h and evaluated expressions of TLR2 signaling-related proteins. TLR2 protein expression in untreated alveolar macrophage from COPD rats was lower than that from control (Fig. 5a). In the assay of *Aspergillus* or Pam3csk4 treatment, there were no significant difference in the levels of AKT and total-Rac1 between the COPD and control groups, but the expression p-AKT and GTP-Rac1 were upregulated. In addition, the growing expressions of TLR2, p-AKT, and GTP-Rac1 in COPD rat group were still lower than those in normal ones under the stimulation of *Aspergillus* or TLR2 agonist. As the phosphorylation of Akt is the downsteam target of PI3K, while the elevation level of GTP-bound form of Rac1 also suggests the activation of Rac1, our data thus indicated that the activation of TLR2/PI3K signaling pathway was impaired in COPD rats in response to *Aspergillus* in a Rac1-dependent manner (Fig. 5b–d; *P < 0.05, #*P* < 0.01).

## DISCUSSION

Although clinical evidence suggested that the incidence of invasive pulmonary aspergillosis (IPA) in the context of COPD is increasing, the mechanisms underlying this association are still poorly understood. In this report, we have demonstrated that CS-exposure substantially attenuated the clearance of *Aspergillus*. In addition, the defect in phagocytosis of *Aspergillus* and inflammatory cytokines release in AMs were observed in COPD rats, which might be associated with impairment of TLR2 activation in AMs. These results provide new insight into the mechanisms by which CS-exposure compromises pulmonary host defense against *Aspergillus* infection.

In this study, however, even with the weaker scavenging of *Aspergillus*, COPD rats did not develop invasive aspergillosis in the lung or any other organs, which suggested that being different from immunocompromised host, COPD rats still have relatively normal defense capability to *Aspergillus*. But the attenuated clearance of *Aspergillus* may result in easier spore colonization in lower respiratory tract, which increases the risk of aspergillosis in the host.

AMs are the sentinel cells of the lung, patrolling the airways to remove any inhaled particles or pathogens [16]. Failure of this innate response could lead to pulmonary damage and persistent infection. AMs from COPD patients phagocytose fewer apoptotic epithelial cells compared with nonsmokers and smokers without COPD [17, 18]. The present study observed a very clear defect in the phagocytic response of AMs from COPD rats to *Aspergillus* conidia. In the BAL fluid from COPD patients, an increase of proinflammatory cytokines and chemokines including TNF-α and IL-8 has been reported, and these mediators may play an important role in establishing and maintaining the inflammatory condition [19]. Cigarette exposure can cause LPS-independent TLR4 activation in AMs, leading to mediator production [20]. Therefore, it is well understood that we observed relatively higher basic levels of TNF-α and MIP-2 in the supernatant of AMs prior to conidia challenge in COPD rats. With the challenge of conidia, elevated levels of proinflammatory cytokines including TNF-α, MIP-2, and IL-1β were observed in two groups, but more significantly in normal rats. Proinflammatory cytokines secreted by AMs play important role in further activation of AMs. Although CS-exposure resulted in some degree of AMs activation, when stimulated with conidia, AMs from COPD rats may not be activated properly, leading to insufficient secretion of inflammatory cytokines. The phagocytic dysfunction mainly displayed as decline of phagocytic rate, which suggested that part of the AMs cannot be normally activated.

IL-10 is an important anti-inflammatory cytokine and can inhibit aggressive inflammatory response. COPD patients combined with IPA usually exhibit severe wheezing. Although the mechanism is not clear, we speculate that it may be associated with allergic reactions. Previous studies had shown that IL-10 played an important role in the inhibition of *Aspergillus* induced allergic reactions in ABPA [21]. In this study, we observed lower secretion of IL-10 in COPD rats, which may partly relate to the wheezing in COPD patients with IPA.

Toll-like receptors (TLRs) are the most important pathogen recognition receptors (PRRs) on macrophages which can activate innate immunity. As a highly conserved family of receptors, they recognize common protein and DNA pattern motifs in microbial pathogens and initiate signaling events related to cytokines production and T cell and DC maturation [22, 23]. Recent studies have shown that TLR2 is involved in recognition of *A. fumigates* [24]. Our data demonstrated reduced expression of TLR2 on AMs of COPD rats before the challenge of *Aspergillus* spores, which might suggest lower proportion of AMs in immune surveillance system of COPD group. Under normal circumstances, the PRRs on AMs will be upregulated with the stimulation of pathogens or some inflammatory cyctokines. The upregulation in both groups was observed. The reason of impaired defense function might partly lie in the regulation of TLR2 on AMs. On d3 after inoculation, the proportion of TLR2-positive cells increased by 50.4 ± 14.6 % in control group, but the COPD group was only 28.0 ± 4.1 %. Factorial analysis results showed different trend of upregulation and downregulation of TLR2 in two groups. The upregulation of TLR2 in control group was faster than that in the COPD group. On d14, the percentages of TLR2-positive cells in COPD rats were higher than those in control rats, which may be associated with continued stimulation of large number of *Aspergillus* spores in the alveolar.

After recognition of microbial products, TLRs may recruit signal moleculars, such as PI3K. Small molecule enzyme Rac1 is one of Rho GTP enzyme super family members, which participates in the regulation of phagocytosis through activating actin cytoskeletal rearrangements [25, 26]. In some cell types, such as CEF cells, Rac1 is a downstream target of the PI3K/Akt signaling cascade, and inactivation of PI3K or Akt was sufficient to suppress Rac1 activation induced by integrin-linked kinase [27]. Therefore, we assessed whether Rac1 is also implicated in the TLR2 signaling pathway by analyzing activation of Akt. Our results show that during *Aspergillus fumigates* or Pam3csk4 stimulation, TLR2 protein expression level was significantly decreased in the COPD rats comparing to that in control, and the levels of p-AKT and GTP-Rac1 were also significantly reduced. This is consistent with the recent report that TLR-2-MyD88-dependent signaling-mediated phagocytosis was dependent on PI3K and Rac1 activation during Mlisteria monocytogenes infection by murine macrophages [28]. Similar finding also demonstrated that stimulation of TLR4 with LPS leads to activation of PI3K and small GTPases in the cell line RAW264.7. TLR4-induced activation of Cdc42 and Rac appears to be independent of MyD88 [29]. Even though, the limitation in our study still exists, which involves validating the effect of TLR2 activation by agonist in the prevention and treatment of *Aspergillus* in individuals with COPD. Additionally, the impairment of adaptive immune response in COPD rats and roles of T-lymphocytes should be further investigated.

In conclusion, we have demonstrated that CS-exposure impairs pulmonary clearance of *A. fumigatus*, which in turn leads to the deficiency of AMs function, including delayed upregulation of TLR2. Given the important role of TLR2 in maintaining the pathogen defense of macrophage, further studies regarding to the molecular mechanism of the expression of TLR2 receptor in COPD rats could lead to more effective treatment regimens of IPA in COPD patients.
